# Dioximate- and Bis(salicylaldiminate)-Bridged Titanium and Zirconium Alkoxides: Structure Elucidation by Mass Spectrometry

**DOI:** 10.1002/cplu.201300014

**Published:** 2013-02-27

**Authors:** Christian Maurer, Ernst Pittenauer, Michael Puchberger, Günter Allmaier, Ulrich Schubert

**Affiliations:** [a] Institute of Materials Chemistry, Vienna University of Technology1060 Vienna (Austria), Fax: (+43) 1-58801-15399 E-mail: Ulrich.Schubert@tuwien.ac.at; [b] Institute of Chemical Technologies and Analytics, Vienna University of Technology1060 Vienna (Austria)

**Keywords:** alkoxides, bridging ligands, mass spectrometry, structure elucidation, titanium

## Abstract

The treatment of titanium alkoxides with 1,5-pentanedioxime or 2,5-hexanedioxime resulted in the formation of complexes [{Ti**L**(OR)_2_}_2_] in which the dioximate ligands (**L**) bridge a dimeric Ti_2_(μ_2_-OR)_2_ unit. The structures of the complexes were determined by single-crystal structure analysis, ESI mass spectrometry, and 1D and 2D solution NMR spectroscopy. In contrast, the treatment of titanium alkoxides with dioximes bearing cyclic linkers, such as cyclohexyl or aryl groups, resulted in insoluble polymeric compounds. The treatment of various bis(salicylaldiminates) with titanium and zirconium alkoxides resulted in compounds with the same composition [{Ti**L**(OR)_2_}_2_], in which, however, two monomeric Ti(OR)_2_ units are bridged by the ligands **L**. The two structural possibilities can be distinguished by low-energy collision-induced dissociation owing to their different fragmentation patterns.

## Introduction

Metal alkoxides are common precursors for sol–gel processing. Modification with bidentate organic ligands, such as β-diketonates, β-ketoesterates, carboxylates, aminoalcoholates, or oximates, lowers their reaction rates and offers the possibility of introducing functional organic groups for the formation of inorganic–organic hybrid materials.^1^ The bidentate ligands are retained largely during sol–gel processing.

Bifunctional ligands Y–X–Y (Y=bidentate coordinating group, X=spacer) have been used rarely for the modification of metal alkoxides. They can be chelating or bridging and result in either polymers of the type [(RO)_*n*_M–Y–X–Y]_∞_ or cyclic compounds [(RO)_*n*_M–Y–X–Y]_*m*_. Such metal alkoxide derivatives offer the possibility of obtaining structured metal oxides after sol–gel processing, similar to alkoxysilane derivatives (RO)_3_Si–Y–Si(OR)_3_.

**Scheme 1:**
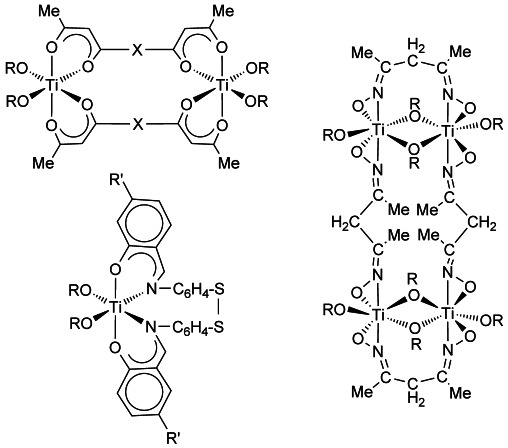
The structures of [(Ti{O*i*Pr}_2_{bis(diketonate)})_2_] (above left, various groups X), [Ti(O*i*Pr)_2_{bis(salicylaldiminate)}] (below left), and [Ti_4_(O*i*Pr)_8_(ON=CMe–CH_2_–CMe=NO)_4_] (right).

A few metal alkoxide derivatives with bifunctional ligands have been reported, but systematic studies were only performed in a few cases. Polymeric structures were previously only obtained by reaction of titanium and zirconium alkoxides with diamines.^2^ These adducts are, however, not suitable for sol–gel processing because of the hydrolytic instability of the Ti–N bond. The tetrameric complex shown in Scheme 1 was obtained from 2,4-pentanedioxime, in which the dioximate ligands both bridge dimeric titanium alkoxide units and interconnect two of the dimeric units.^3^ In the absence of an additionally coordinating substituent the salicyclaldiminate-substituted derivatives [M(OR)_2_(SA)_2_] (M=Zr, Ti; SA=salicylaldiminate) have the same geometry as the corresponding complexes [M(OR)_2_(β-diketonate)_2_], that is, with the OR groups in positions *cis* to each other.^4^ This structural motif was also found in a complex in which the two SA ligands are connected through a C_6_H_4_–S–S–C_6_H_4_ bridge (Scheme 1, bottom left)^5^ as well as in the hydrolyzed compound [{(salen)TiO}_2_] (salen=*N*,*N*′-ethylenebis(salicylimine)), in which the two Ti atoms are bridged by two oxo groups.^6^ Recently Tzubery et al. reported the monomeric compound [Ti(salen)(OC_6_H_3_Me_2_)_2_] with the aryloxo ligands in positions *trans* to each other.^7^

We recently described reactions of various bis(β*-*diketones) and bis(β-ketoesters) with titanium and zirconium alkoxides, which in each case resulted in cyclic oligomers.^8^ For example, reaction with Ti(O*i*Pr)_4_ resulted in the cyclic dimers [(Ti{O*i*Pr}_2_{bis(diketonate)})_2_] (see Scheme 1, top left); complexes with a higher degree of substitution were additionally formed with Zr(O*i*Pr)_4_.

**Scheme 2:**
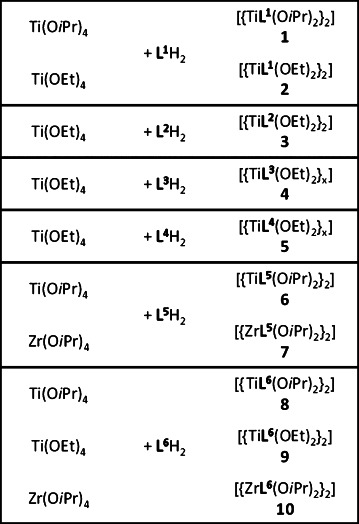
Numbering scheme of the prepared compounds.

The way in which the bifunctional ligands are coordinated and the kind of compounds that are formed depends not only on the nature of the coordinating groups Y and the length and rigidity of the spacer X, but also on the coordination geometry at the metal center. We therefore extended our studies in the current work on reactions of M(OR)_*n*_ (M=Ti, Zr) with dioximes and bis(salicylaldiminates) with various spacer groups X (Scheme 2).3
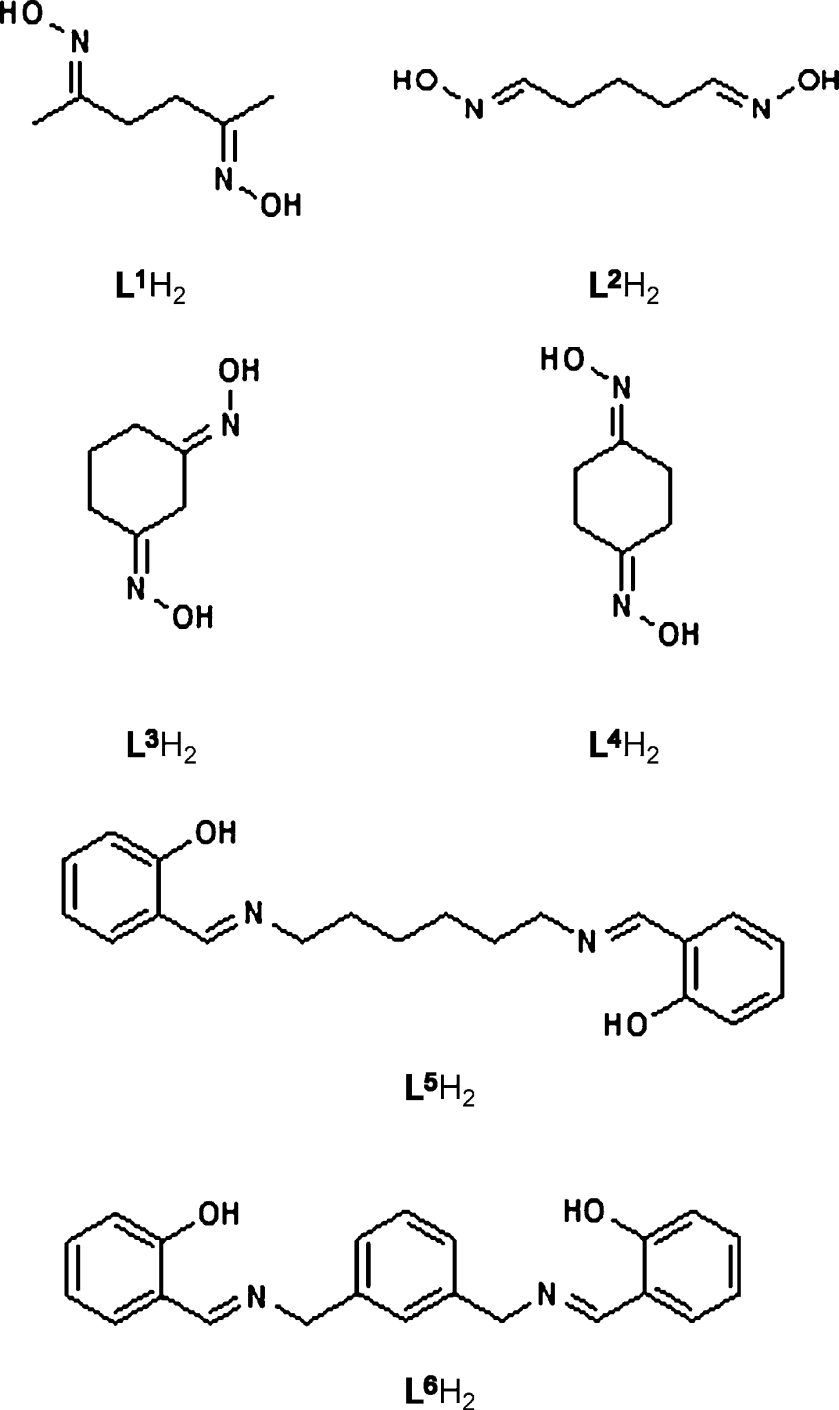


## Results and Discussion

### Alkylene-bridged dioximes

Reaction of various oximes with zirconium or titanium alkoxides mainly resulted in dimeric complexes [{Ti(OR)_2_(ON=CR′R′′)_2_}_2_] (R=*i*Pr, Et) or [{Zr(O*i*Pr)(oximate)_3_}_2_] with two bridging alkoxo groups and two oximate ligands. Contrary to bis(β-diketonate) derivatives, the oximate ligands are positioned *trans* to each other.^3^, ^9^ Reaction of one molar equivalent of Ti(O*i*Pr)_4_ with one equivalent of **L^1^**H_2_ in 1,2-dichloroethane or of Ti(OEt)_4_ with **L^1^**H_2_ or **L^2^**H_2_ in ethanol resulted in colorless solutions, from which **1** and **3** crystallized after slow evaporation of the solvent [Eq. (1) for compound **3**]. Compound **2** did not crystallize but was characterized by mass spectrometry (see below).1
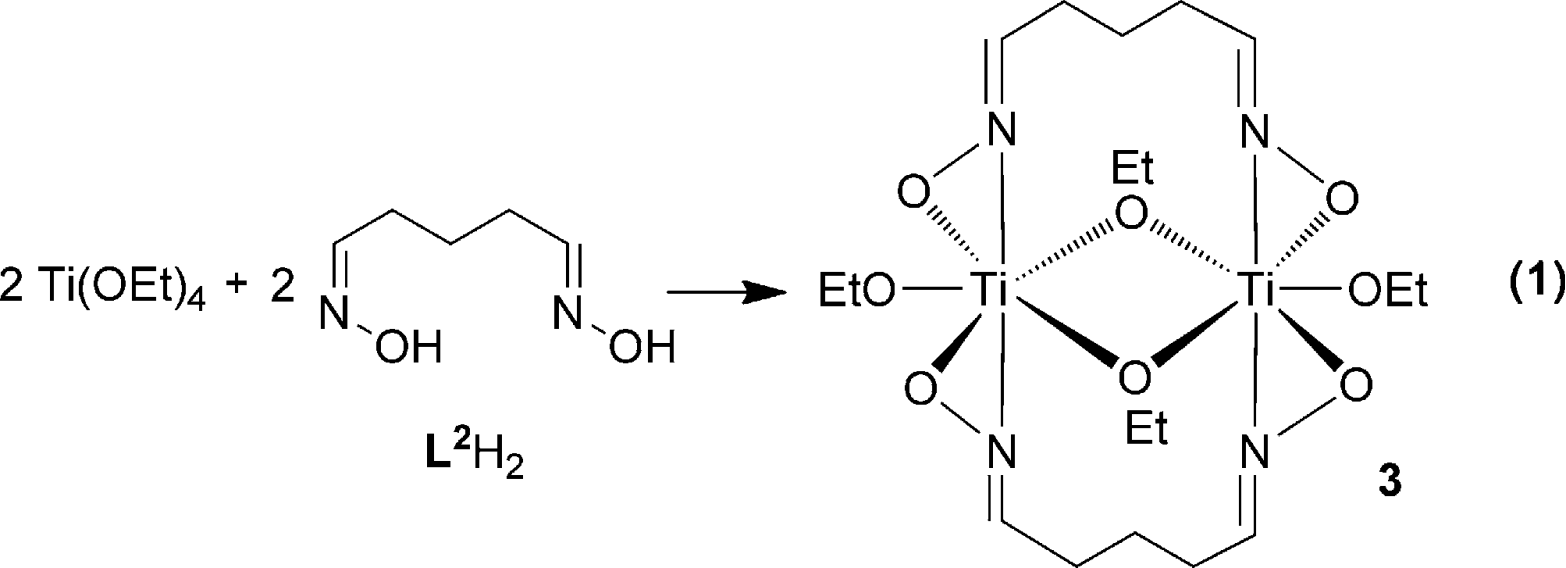


**Figure 1:**
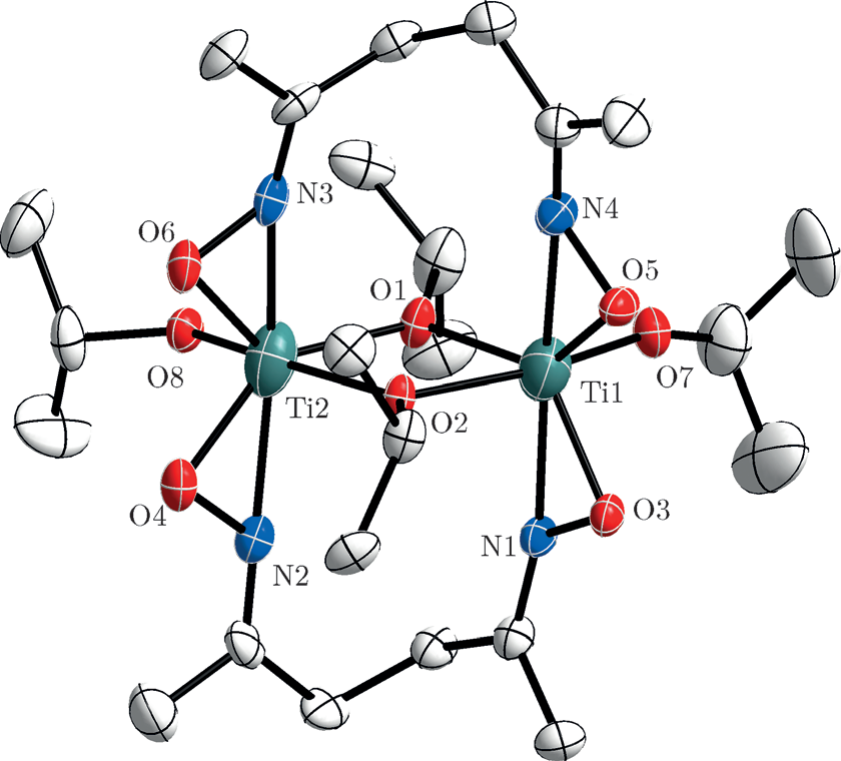
Molecular structure of [{Ti**L^1^**(O*i*Pr)_2_}_2_] (**1**). The hydrogen atoms were omitted for clarity. Selected bond lengths [pm] and angles [°]: Ti1–O1 204.6(3), Ti1–O2 203.0(3), Ti1–O3 196.5(3), Ti1–O5 195.4(3), Ti1–O7 179.9(4), Ti1–N1 203.2(4), Ti1–N4 216.9(4), O1–Ti1–O2 74.92(1), Ti2–O1 203.8(3), Ti2–O2 206.2(2), Ti2–O4 192.3(4), Ti2–O6 196.3(3), Ti2–O8 181.2(3), Ti2–N2 214.5(4), Ti2–N3 206.1(4), O1–Ti2–O2 74.41(1), O1–Ti1–O7 91.75(1), O2–Ti1–O7 166.67(1), O3–Ti1–O7 98.56(1), O3–Ti1–O5 85.40(1), O5–Ti1–O7 97.94(2), N1–Ti1–N4 161.23(1), O1–Ti2–O8 92.80(1), O2–Ti2–O8 166.19(2), O4–Ti2–O6 86.54(1), O4–Ti2–O8 93.88(1), O6–Ti2–O8 97.00(1), N2–Ti2–N3 162.89(1).

**Figure 2:**
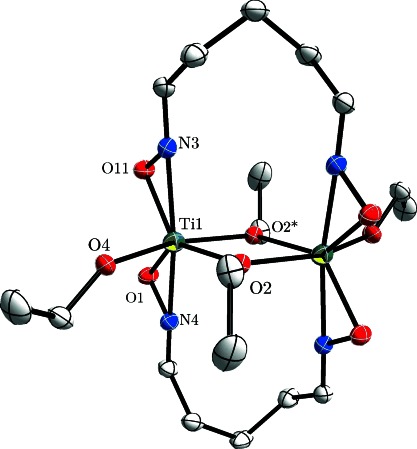
Molecular structure of [{Ti**L^2^**(OEt)_2_}_2_] (**3**). The hydrogen atoms were omitted for clarity. The asterisk denotes inversion-related atoms. Selected bond lengths [pm] and angles [°]: Ti1–O1 195.63(14), Ti1–O2 204.75(13), Ti1–O2* 202.78(13), Ti1–O4 180.68(14), Ti1–O11 195.30(14), Ti1–N3 209.0(2), Ti1–N4 209.2(2), O1–Ti1–O4 98.09(6), O1–Ti1–O11 85.67(7), O2–Ti1–O2* 71.61(6), O2–Ti1–O4 92.07(6), O2*–Ti1–O4 162.46(6), O4–Ti1–O11 100.09(6), N3–Ti1–N4 164.48(7).

The structures (Figures 1 and 2) of **1** and **3** were determined by single-crystal XRD. Both compounds form dimeric complexes with two bridging dioximate groups and two μ_2_-OR groups. Contrary to the structurally related compounds [{Ti(OR)_2_(ON=CHR′)_2_}_2_],^9^, ^3^ neighboring oximate moieties at the two titanium atoms in **1** and **3** are connected through the (CH_2_)_*n*_ spacer. Contrary to [Ti_4_(O*i*Pr)_8_(ON=CMe–CH_2_–CMe=NO)_4_] (Scheme 1, right), both dioximate ligands bridge the same Ti_2_(O*i*Pr)_4_ unit. This difference is probably due to the smaller strain of the more flexible **L^1^** or **L^2^** ligands if both bridge titanium atoms of the same dimeric unit. There is a noteworthy difference between the cyclic compounds [(Ti{O*i*Pr}_2_{bis(diketonate)})_2_]^8^ (Scheme 1) and [(Ti{O*i*Pr}_2_{bis(dioximate)})_2_] [Eq. (1)]. The latter contain two μ_2_-O*i*Pr and two terminal O*i*Pr groups and the former only terminal O*i*Pr groups. In [(Ti{O*i*Pr}_2_{bis(diketonate)})_2_], μ_2_-O*i*Pr groups would lead to a much more crowded coordination sphere at the titanium centers, owing to the larger bite angle of the β-diketonate groups than the oximate groups.

The Ti–O and Ti–N bond lengths and selected angles are in a comparable range as in the previous investigated compounds [{Ti(O*i*Pr)_2_(ON=CHR′)_2_}_2_].^3^, ^9^ The alkoxo bridges are slightly asymmetric, with Ti–O_bridging_ bond lengths ranging from 202.5 to 206.2 pm. The oximate groups bonded to the same titanium center are nearly coplanar.

**Figure 3:**
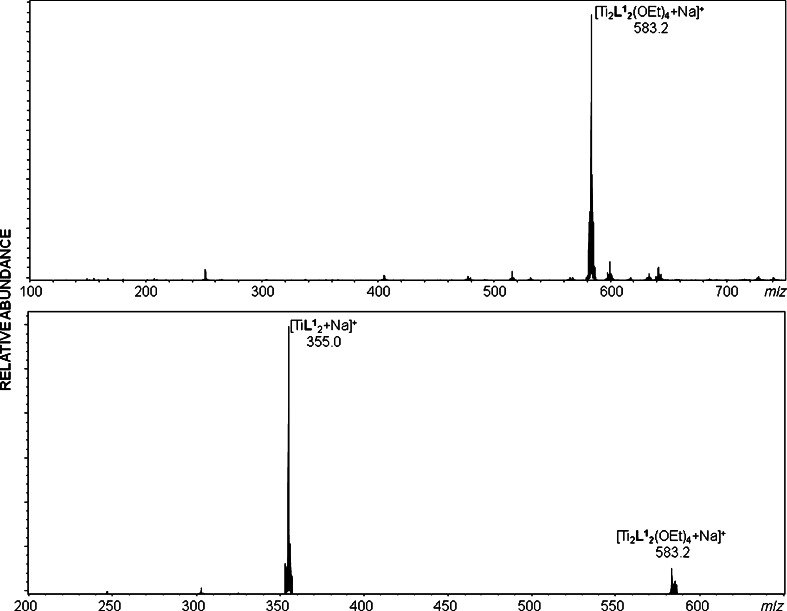
Positive-ion ESI mass spectrum (top) and low-energy CID (MS/MS) spectrum (bottom) of [{Ti**L^1^**(OEt)_2_}_2_] (**2**).

**Scheme 3:**
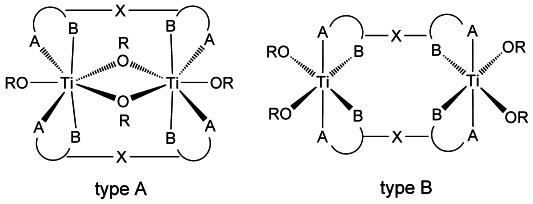
Two structure types of compounds [{Ti**L**(OR)_2_}_2_] (**L**=bridging bifunctional ligand with chelating groups A–B).

As compound **2** did not crystallize, electrospray ionization mass spectrometry (ESI-MS) was used for characterization. We have recently shown for cyclic [(Ti{O*i*Pr}_2_{bis(diketonate)})_2_] compounds that ESI-MS is a suitable method for structure elucidation of such compounds.^8^ The mass spectrometric investigations showed that **2** and **3** have the same structure. The mass spectra of **2** (Figure 3) and **3** showed the intact sodiated molecule ion [Ti_2_**L**_2_(OEt)_4_+Na]^+^ at *m*/*z* 583.2 (calcd 583.2) [**2**+Na]^+^ and at *m*/*z* 555.2 (calcd 555.1) [**3**+Na]^+^. No protonated molecules were observed. Low-energy collision-induced dissociation (CID) of the precursor ion, that is, sodiated molecule, resulted in a clear fragmentation to one specific fragment ion [Ti**L_2_**+Na]^+^. This contrasts with [(Ti{O*i*Pr}_2_{bis(diketonate)})_2_],^8^ in which a fragment ion [Ti**L**(O*i*Pr)_2_+Na]^+^ was detected. Whereas the bis(β-diketones) formed metallacycles with two bridging ligands and four terminal O*i*Pr groups (type B in Scheme 3), the dimeric dioximate structures contain two μ_2_-OR and two terminal alkoxo groups (type A in Scheme 3). The μ_2_-OR groups apparently promote the cleavage of a Ti(OEt)_4_ unit after the fragmentation. The different fragmentation pattern of the MS/MS experiment thus allows a clear distinction between type A and type B titanium complexes. The mass spectrometric investigations of complexes **8** and **9** discussed later in this article prove that the different fragmentation pathway is not caused by the use of Ti(OEt)_4_ instead of Ti(O*i*Pr)_4_.

The covered *m*/*z* range was increased to higher values to be able to monitor high molecular mass species and to eliminate the formation of oligomeric or polymeric compounds. In both cases only the molecular-ion peak was present in the mass spectrum. Increasing the Ti(O*i*Pr)_4_/dioxime ratio resulted in the same sodiated molecular species, while the intensity of [Ti(O*i*Pr)_4_+Na]^+^ at *m*/*z* 307.1 (calcd 307.1) increased. Thus, the formation of mono-substituted titanium–alkoxo–oximate derivatives can be excluded.

Solution NMR spectroscopy also proved the coordination of both oximate groups to the titanium centers. The shift of the C=N signal in the ^13^C NMR spectra changed from *δ*=155.3 and 150.5 ppm in the starting oxime to *δ*=144.8/146.0 and 139.4 ppm in compounds **1**–**3**, which is in agreement with previously investigated titanium–alkoxo–oximate derivatives. Resonances for the CH/CH_2_ groups of two different alkoxo groups were observed in all ^1^H NMR spectra, at *δ*=4.47/3.37 ppm for **1**, *δ*=4.35/3.91 ppm for **2**, and *δ*=4.34/3.64 ppm for **3**. Similar observations were also made in the corresponding ^13^C NMR spectra. NMR spectroscopy also revealed two different signals for the CH_3_ group of **L^1^** in compounds **1** and **2**.

**Figure 4:**
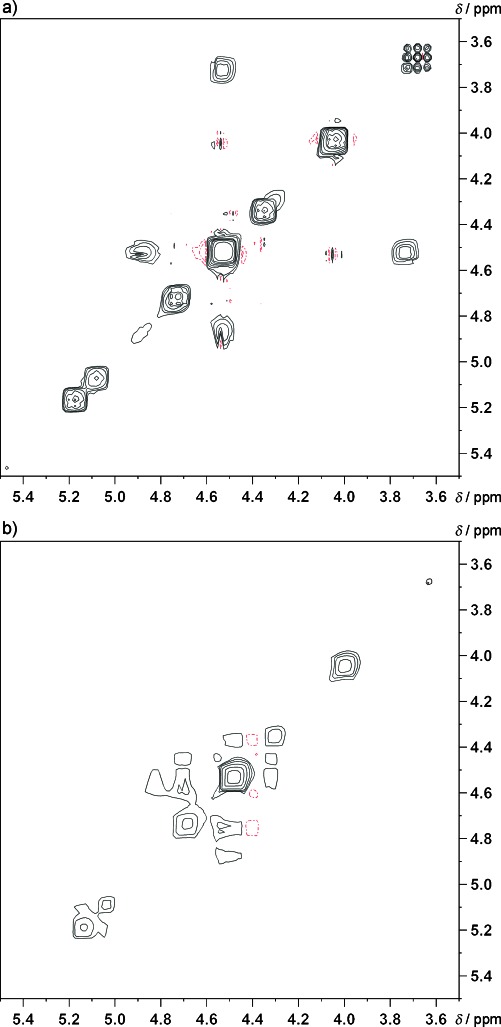
EXSY spectrum of **1** in the CH region at (a) room temperature and (b) −60 °C.

Because of its better solubility, compound **1** was investigated by 2D NMR spectroscopy. Seven different CH signals were observed in the HSQC spectrum at room temperature (Figure 4). This can be explained by different conformations of the nine-membered ring, formed by a dioximate and a bridging O*i*Pr ligand (i.e., –Ti–O_br_–Ti–N–C–C–C–C–N–), which leads to independent O*i*Pr signals.

**Figure 5:**
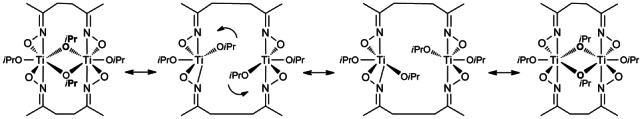
Possible mechanism of active O*i*Pr exchange in **1**.

The exchange spectroscopy (EXSY) spectrum at room temperature (Figure 4a) showed that the compound is highly dynamic. Exact interpretation is difficult, as the methylene signals are not split. Only two to three signals were observed, although four signals are expected for this AB system. Two exchange signals at *δ*=3.7/4.5 and 4.5/4.85 ppm were observed. The resonance at *δ*=3.7 ppm was assigned to free 2-propanol; the first set of exchange signals is therefore caused by the exchange of residual 2-propanol and a terminal O*i*Pr group. The second exchange signal (*δ*=4.5/4.85 ppm) was attributed to an exchange between different O*i*Pr groups. One possible explanation is active exchange between terminal and bridging O*i*Pr groups. A possible mechanism is shown in Figure 5; opening of the O*i*Pr bridges would result in a dimer only bridged by the dioximate ligands. Rotation of the metal alkoxide moieties and recombination of the bridges would result in exchanged alkoxo groups.

The second possibility is a passive exchange in which the bridging O*i*Pr groups follow the dynamics of the alkylene chain connecting the oximate groups. If the conformations of the nine-membered ring changes, the bridging O*i*Pr ligands might also be influenced.

**Figure 6:**
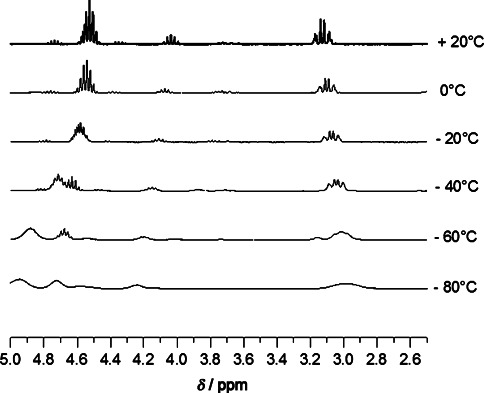
Temperature-dependent ^1^H NMR spectra of **1**.

Temperature-dependent NMR spectroscopy experiments were conducted between +20 and −80 °C (Figure 6). The methine signal of the O*i*Pr group and that of the N–CH_2_ group in the ^1^H NMR spectrum were broad. When lowering the temperature, the methine signal at *δ*=4.47 ppm broadened into two independent signals, that is, more conformers can be distinguished. The COSY spectrum at −60 °C revealed eleven to twelve independent CH signals, which proved the existence of at least three different conformers at low temperature. The previously observed exchange signals disappeared in the EXSY spectrum at −60 °C (Figure 4b). The dynamics of both processes were therefore minimized.

A similar solution structure is postulated for **2** and **3** based on NMR spectroscopy and MS measurements.

### Cyclohexanedioximes

Bridging of the titanium atoms in an alkoxo-bridged dimeric Ti_2_(OR)_4_ unit (type A in Scheme 3) is only possible if the spacer between the two coordinating units is flexible enough to adjust to the Ti–Ti distance in this unit, which is determined by the geometry of the central Ti_2_O_2_ ring. Stiffening of the spacer could therefore induce the compounds to adopt a different structure. Reaction of one molar equivalent of Ti(OEt)_4_ with 1,3- or 1,4-cyclohexyldioxime resulted in the barely soluble, amorphous compounds **4** and **5**. Both were only reasonably soluble in ethanol at elevated temperature. As discussed below, there is evidence, however, that the compounds degrade upon dissolution in ethanol. The solid-state ^13^C NMR spectrum of **4** showed a clear shift of the oximate carbon to *δ*=143.0 ppm, which is proof of coordination of the oximate groups.^3^, ^9^ The spectrum also revealed the presence of OEt groups by the signal at *δ*=68.7 ppm assigned to the OCH_2_ groups. The shift of the oximate nitrogen in the ^15^N NMR spectrum from *δ*=278.8 ppm in **L^3^**H_2_ to *δ*=274.0 ppm in **4** confirmed the coordination of the oximate groups.

ESI- and MALDI-MS measurements (the latter not described here in detail) of solutions of **4** and **5** in ethanol did not result in signals for compounds of reasonable composition related to the charged molecule, but only ions representing **L^3^**H_2_ and titanium alkoxides were observed. A possible explanation for this is that the compounds do not dissolve in ethanol but are instead degraded in solution.

The very low solubility of products **4** and **5** indicated a polymeric structure. A glass transition was observed by differential scanning calorimetry (DSC) at approximately 80 °C for **4**, which would be in line with a polymeric structure. No glass transition was observed for **5** and therefore equilibria between oligomeric and polymeric structures must also be considered. A polymeric structure could be based on either the type A or the type B motif (Scheme 3), that is, with or without bridging OR ligands.

Reaction of titanium alkoxides with aryl-bridged dioximates results in compounds with similar properties and therefore analogous polymeric structures are proposed.

### Bis(salicylaldimines)

Reaction of M(OR)_4_ (M=Ti, Zr; R=*i*Pr, Et) with one molar equivalent of the bis(salicylaldimines) **L^5^**H_2_ or **L^6^**H_2_ in 1,2-dichloroethane or dichloromethane resulted in the yellowish solids **6**–**10**, which could not be crystallized [Eq. (2)].2
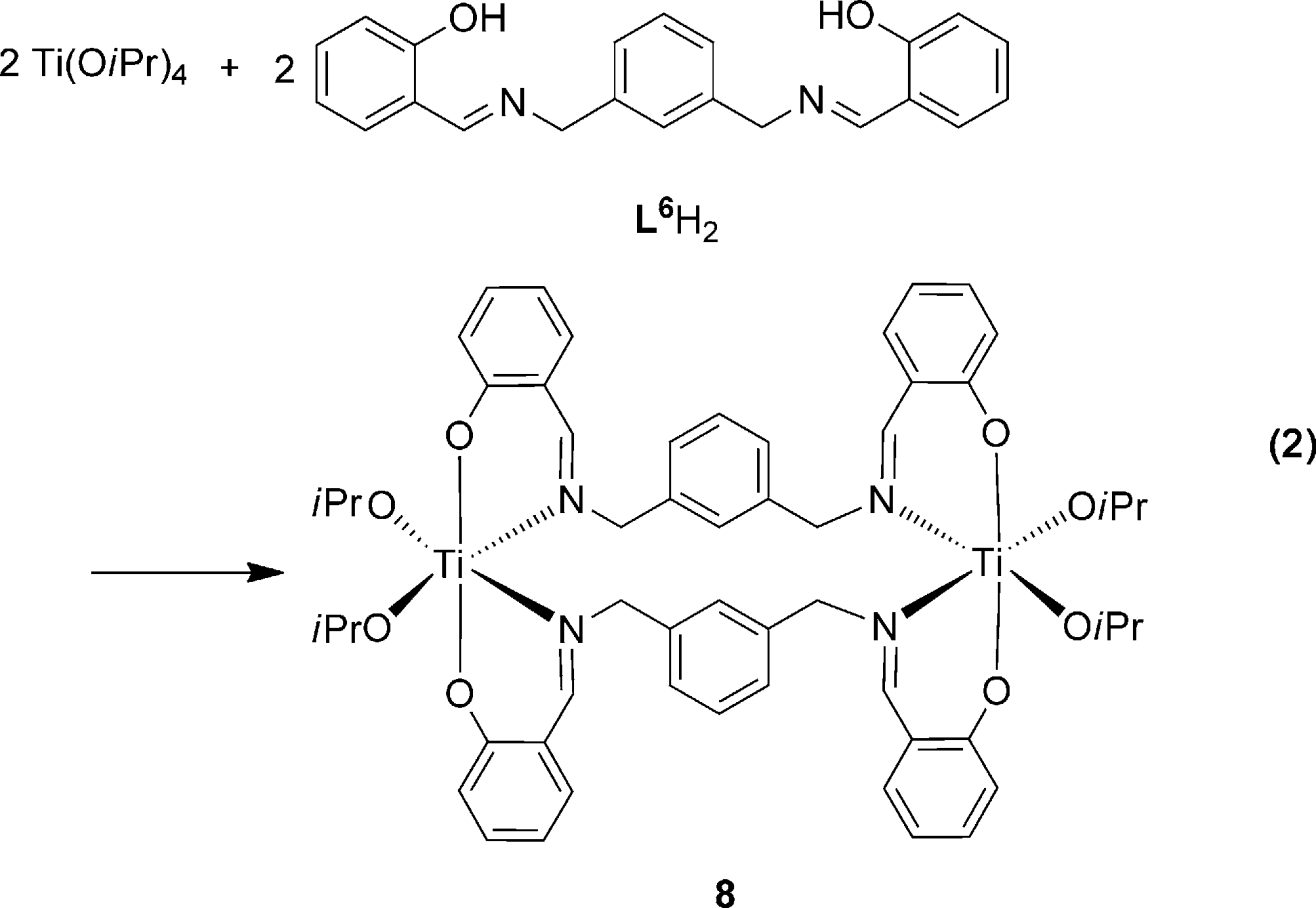


**Figure 7:**
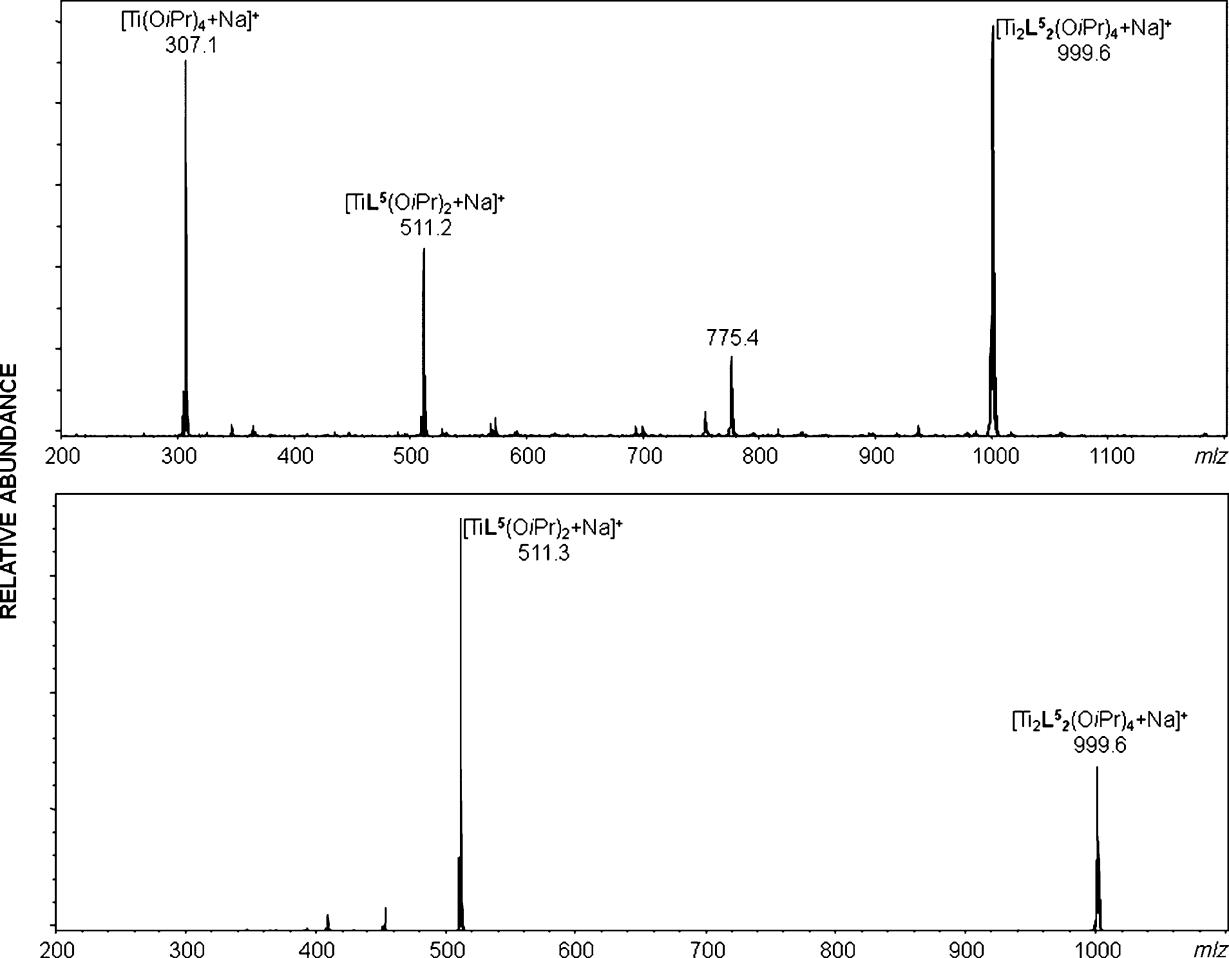
Positive-ion ESI mass spectrum (top) and low-energy CID (MS/MS) spectrum (bottom) of [{Ti**L^5^**(O*i*Pr)_2_}_2_] (**6**).

Information on the composition of **6**–**10** was gained from ESI-MS. An intense molecular-ion peak with the composition [{M**L**(OR)_2_}_2_] was observed for compounds **6**–**10**. The titanium species in **6**, **8**, and **9** were detected as the sodiated molecule, whereas the zirconium compounds **7** and **10** were detected as the sodiated and chloro-adduct molecules. As an example of all the above-mentioned compounds, the positive-ion ESI mass spectrum of **6** (Figure 7) is discussed in detail. The sodiated molecule detected at *m*/*z* 999.6 (calcd 999.4 for [Ti_2_**L^5^**_2_(O*i*Pr)_4_+Na]^+^). The calculated molecular mass corresponds to a dimeric structure with two bridging bis(salicylaldiminate) ligands. MS/MS (low-energy CID) experiments were conducted to determine whether the compound is of type A or B (Scheme 3). A single peak at *m*/*z* 511.2 (calcd 511.2) in the MS/MS spectrum was assigned to [Ti**L^5^**(O*i*Pr)_2_+Na]^+^. Similar fragmentation was previously observed for various bis(β-diketonate)-substituted titanium alkoxide derivatives.^8^ This fragmentation pathway indicated a type B structure (Scheme 3). The MS/MS measurement also revealed that the signal at *m*/*z* 511.3, which appeared also in the full-scan mass spectrum (*m*/*z* 511.2), was only caused by in-source fragmentation and was not due to another compound (or contamination) in the solution. The peak appearing at *m*/*z* 775.4 (calcd 775.3) was attributed to [(Ti**L^5^**_2_)*i*PrOH+Na]^+^, an ion corresponding to the monomeric titanium complex with two coordinated bis(salicylaldiminate) ligands. As this signal did not appear at all in the MS/MS spectrum (lower spectrum in Figure 7), it was no fragment ion, but instead a byproduct of the reaction. Corresponding signals did not appear in the mass spectra of compounds **7**–**10**. Otherwise the mass and CID spectra were the same, also for zirconium compounds **7** and **10**, implying that all compounds have the same structure. A change of the M(OR)_4_/bis(salicylaldimine) ratio resulted in the same molecular ion peaks.

Analogous structures were previously reported for [{Pt(**L^5^**)}_2_] and [{Cu(**L^5^**)}_2_] complexes (metal ion instead of the M(OR)_2_ entity),^10^, ^11^ whereas a polymeric structure was proposed for Ni^II^ complexes of C_*n*_–bis(salicylaldiminates) (*n*=6–12).^12^

The solution properties of **6**–**10** were investigated by NMR spectroscopy experiments. A clear shift of the C*H*=N proton from *δ*=8.46 to 7.92–7.63 ppm was observed for all compounds, and the chemical-shift difference of the *C*H=N and *C*_aryl_–O signals in the ^13^C NMR spectrum became smaller. Both observations are clear evidence of coordination of the salicylaldiminate groups. All spectra showed only one signal for CH/CH_2_ groups of terminal alkoxo groups, in line with the conclusions from the ESI-MS spectra.

## Conclusion

Reactions of titanium or zirconium alkoxides with dioximes or bis(salicylaldimines) resulted in complexes of the type [{Ti**L**(OR)_2_}_2_] (**L**=bridging dioximate or bis(salicylaldiminate) ligands), when the spacer between the coordinating groups was flexible enough. Two structural possibilities (Scheme 3) were observed that could be distinguished clearly by ESI-MS and especially MS/MS experiments in the low-energy CID mode. Type A complexes were formed with dioximate ligands, in which the ligands bridge a Ti_2_(μ_2_-OR)_2_ unit. In contrast, the bis(salicylaldiminate) ligands bridge two independent M(OR)_2_ (M=Ti, Zr) moieties (type B complexes). The latter structure type was previously observed also for bis(β*-*diketonate)-substituted titanium isopropoxide derivatives.^8^

When the spacer between the two oximate groups was stiffened, that is, when titanium alkoxides were treated with 1,3- or 1,4-cyclohexyldioxime, insoluble compounds with a glass transition were formed, which appear to have a polymeric structure.

## Experimental Section

All operations were carried out under moisture- and oxygen-free argon using standard Schlenk or glovebox techniques. Ti(OEt)_4_ (Aldrich), Ti(O*i*Pr)_4_ (Aldrich, 97 %), Zr(O*i*Pr)_4_⋅*i*PrOH (ABCR), and all chemicals for ligand syntheses were used as received. Solvents were dried and purified by standard techniques. CDCl_3_ (Aldrich, 99.9 %), CD_2_Cl_2_ (euriso-top, 99.5), C_6_D_6_ (euriso-top, 99.5 %), [D_6_]DMSO (Aldrich, 99.6 %), and [D_8_]toluene (euriso-top, 99.6 %) used for NMR spectroscopy experiments were degassed and dried over 3 Å molecular sieves. No yields were determined for complexes that could not be crystallized. Characterization was done directly out of the solution or from the dried residues.

### Characterization techniques

The ^1^H and ^13^C solution NMR spectra were recorded on a Bruker Avance 250 (250.13 MHz {^1^H}, 62.86 MHz {^13^C}). Samples for solution NMR spectra were taken by dissolving the dried residue in a deuterated solvent without further purification. 2D NMR spectra were performed on a Bruker Avance 300 DPX (300.13 MHz {^1^H}, 75.47 MHz {^13^C}) and measured with Bruker standard pulse programs COSY, HSQC, EXSY (*t*_mix_=1 s), and HMBC (optimized for *J*=140 Hz). Solid-state NMR spectra were recorded on a Bruker Avance 300 instrument equipped with a 4 mm broad-band magic-angle spinning (MAS) probe head operating at 75.4 MHz for ^13^C and 30.4 MHz for ^15^N. The ^13^C and ^15^N NMR spectra were recorded with ramped CP/MAS at a rotor frequency of usually 6–8 kHz.

The ESI-MS measurements were performed on a Bruker Daltonics Esquire 3000^plus^ 3D ion-trap mass spectrometer fitted with an orthogonal ESI ion source and operated in the positive- or negative-ion mode. The spray voltage was maintained at −4 kV, the drying gas temperature was set to 200 °C and all ion and transfer-line source voltages were optimized for maximum molecular-ion transmission (i.e., the sodiated or chloro-adduct molecules). For low-energy CID MS/MS experiments, the isolation width was typically set to 10 Da to cover the entire isotopic distribution of the selected precursor ion. The fragmentation amplitude was manually set to 0.5–1 V to induce abundant product-ion formation. Solutions in pure 2-propanol or in a mixture of chloroform/2-propanol (1:3) at a concentration of 1 mg mL^−1^ were infused by a syringe pump into the ESI-source at a flow rate of 3 μL min^−1^. No sodium chloride was added to the sample solution to enhance formation of adduct ions. All calculated *m*/*z* values of the titanium complexes were based on the naturally most abundant ^48^Ti isotope, and those of the zirconium complexes based on the most abundant ^90^Zr isotope.

The MALDI-MS evaluation (for **4** and **5**) was performed by means of a Shimadzu Kratos Analytical Axima CFR^+^ in the positive-ion mode by applying standard MALDI matrices.

### X-ray structure analyses

Single-crystal X-ray diffraction experiments were performed at 100 K on a Bruker-AXS SMART APEX II diffractometer with a CCD area detector and a crystal-to-detector distance of 5.0 cm using graphite-monochromated Mo_Kα_ radiation (*λ*=71.073 pm). Data were collected with *ϕ* and *ω* scans and 0.5° frame width. The data were corrected for polarization and Lorentz effects, and an empirical absorption correction (SADABS) was applied.^13^ The cell dimensions (Table 1) were refined with all unique reflections. The structures were solved with direct methods (SHELXS97) and refinement to convergence was carried out with the full-matrix least-squares method based on *F*^2^ (SHELXL97) with anisotropic structure parameters for all non-hydrogen atoms.^14^, ^15^ The hydrogen atoms were placed on calculated positions and refined riding on their parent atoms. CCDC 907994 http://www.ccdc.cam.ac.uk/cgi-bin/catreq.cgi(**1**) and 907995 http://www.ccdc.cam.ac.uk/cgi-bin/catreq.cgi(**3**) contain the supplementary crystallographic data for this paper. These data can be obtained free of charge from The Cambridge Crystallographic Data Centre via http://www.ccdc.cam.ac.uk/data_request/cif.

**Table 1:** Crystallographic parameters of 1 and 3

	1	3
formula	C_24_H_48_N_4_O_8_Ti_2_	C_18_H_36_N_4_O_8_Ti_2_
*M*_r_	616.6	532.3
*T* [K]	100	100
crystal system	monoclinic	monoclinic
space group	*Cc*	*P*2_1_/*c*
*a* [pm]	989.40(9)	1548.15(5)
*b* [pm]	3062.1(3)	1167.11(4)
*c* [pm]	1072.13(9)	1363.70(5)
*β* [°]	106.4530(10)	96.862(2)
*V* [pm^3^×10^6^]	3115.2(5)	2446.37(15)
*Z*	4	4
*ρ*_calcd_ [g m^−3^]	1.314	1.445
*μ* [mm^−1^]	0.560	0.701
crystal size [mm]	0.40×0.40×0.20	0.43×0.31×0.19
*θ* range [°]	1.33–24.98	1.32–30.62
reflns collected/unique	8442/4492	26 049/7522
data/parameters	4492/355	7522/293
GOF on *F*^2^	0.712	0.703
*R* [*I*>2*σ*(*I*)]	0.045	0.042
*wR*2	0.144	0.113
largest diff. peak/hole [e Å^−3^]	0.540/−0.445	0.653/−0.975

### Synthesis of ligands

The dioximes **L^1^**H_2_, **L^2^**H_2_, **L^3^**H_2_, and **L^4^**H_2_ were synthesized by a modification of the method described by Bousquet.^16^ In a typical procedure hydroxylamine hydrochloride (14.5 g, 209 mmol) was dissolved in deionized water (50 mL) and cooled to 0 °C. The corresponding diketone/dialdehyde (87 mmol) was added dropwise. After 30 min of stirring at room temperature a solution of potassium carbonate (14.4 g, 104 mmol) in deionized water (25 mL) was added. The solution was stirred at ambient temperature overnight. The formed precipitate was removed by filtration and recrystallized from EtOH or *i*PrOH.

**L^1^**H_2_: yield: 75 mmol (86 %); ^1^H NMR (250 MHz, [D_6_]DMSO, 25 °C, TMS): *δ*=10.3 (s, 2 H; N–O*H*), 2.25 (s, 4 H; CH_2_C*H_2_*CN), 1.69 ppm (s, 6 H; C*H_3_*CN); ^13^C NMR (62.86 MHz, [D_6_]DMSO, 25 °C, TMS): *δ*=155.3 (CH_3_*C*NCH_2_), 32.5 (CN*C*H_2_CH_2_), 13.5 ppm ((*C*H_3_)_2_CN).

**L^2^**H_2_: yield: 78 mmol (90 %); ^1^H NMR (250 MHz, [D_6_]DMSO, 25 °C): *δ*=10.77 (s, 2 H; N–O*H*), 6.64 (s, 2 H; C*H*=N), 2.20 (t, 4 H; CH_2_C*H_2_*CN), 1.54 ppm (m, 2 H; CH_2_C*H*_2_CH_2_); ^13^C NMR (62.86 MHz, [D_6_]DMSO, 25 °C, TMS): *δ*=150.5 (*C*HNCH_2_), 25.7 (CN*C*H_2_CH_2_), 23.0 ppm (CH_2_*C*H_2_CH_2_).

**L^3^**H_2_: yield: 84 mmol (97 %); ^1^H NMR (250 MHz, [D_6_]DMSO, 25 °C, TMS): *δ*=10.42 (s, 2 H; N–O*H*), 3.42 (s, 2 H; CNC*H_2_*CN), 2.29 (t, 4 H; CNC*H_2_*CH_2_), 1.62 ppm (m, 2 H; CH_2_C*H_2_*CH_2_); ^13^C NMR (62.86 MHz, [D_6_]DMSO, 25 °C, TMS): *δ*=153.6 ((CH_2_)_2_*C*N), 31.0 (CH_2_*C*H_2_C), 24.4 (C*C*H_2_C), 23.7 ppm (CH_2_*C*H_2_CH_2_); ^13^C CP-MAS NMR: *δ*=152.9 (CH_2_*C*NCH_2_), 31.4 ppm (CH_2_*C*H_2_C, CH_2_*C*H_2_CH_2_, CN*C*H_2_CN); ^15^N CP-MAS NMR: *δ*=278.8 ppm.

**L^4^**H_2_: yield: 85 mmol (98 %); ^1^H NMR (250 MHz, [D_6_]DMSO, 25 °C, TMS): *δ*=10.33 (s, 2 H; N–O*H*), 2.43 (t, 4 H; C*H_2_*), 2.34 ppm (t, 4 H; C*H_2_*); ^13^C NMR (62.86 MHz, [D_6_]DMSO, 25 °C, TMS): *δ*=156.6 ((CH_2_)_2_*C*N), 26.9 (CH_2_*C*H_2_C), 23.9 ppm (CH_2_*C*H_2_C); ^13^C CP-MAS NMR: *δ*=157.8 (CH_2_*C*NCH_2_), 36.5 (CH_2_*C*H_2_C), 24.6 ppm (CH_2_*C*H_2_C); ^15^N CP-MAS NMR: *δ*=278.1, 221.4 ppm (C=N⋅⋅⋅HO–N).

### Synthesis of the complexes

[{Ti**L^1^**(O*i*Pr)_2_}_2_] (**1**): 2,5-Hexanedioxime (95 mg, 0.66 mmol) was dissolved in 1,2-dichloroethane (3 mL) and Ti(O*i*Pr)_4_ (0.2 mL, 0.66 mmol) was added dropwise. The solution was stirred for 30 min at room temperature. Colorless crystals of **1** (152 mg, 75 %) were obtained after two weeks by slow evaporation of the solvent. ^1^H NMR (250 MHz, CDCl_3_, 25 °C, TMS): *δ*=4.47 (m, 2 H; (CH_3_)_2_C*H*), 3.37 (m, 2 H; (CH_3_)_2_C*H*), 3.12 (m, 4 H; CH_2_C*H_2_*CN), 2.12 (m, 4 H; CH_2_C*H_2_*CN), 1.82 (s, 12 H; C*H_3_*CN), 1.28 (d, 12 H; (C*H_3_*)_2_CH), 1.17 ppm (d, 12 H; (C*H_3_*)_2_CH); ^13^C NMR (62.86 MHz, CDCl_3_, 25 °C, TMS): *δ*=144.8 (CH_3_*C*NCH_2_), 77.1/75.9 ((CH_3_)_2_*C*H), 30.5 (CN*C*H_2_CH_2_), 26.2/25.7 ((*C*H_3_)_2_CH), 19.9/17.0 ppm (*C*H_3_CN); IR (ATR): 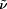
=2967 (C–H), 2918 (C–H), 1658 (C=N), 1428 (C–C), 1360 (C–O–Ti), 1121 (C–O), 995 (C–O), 934, 849, 819 cm^−1^.

[{Ti**L^1^**(OEt)_2_}_2_] (**2**) was synthesized similarly from Ti(OEt)_4_ with ethanol as the solvent. ^1^H NMR (250 MHz, CDCl_3_, 25 °C, TMS): *δ*=4.35 (q, 4 H; CH_3_C*H_2_*), 3.91 (q, 4 H; CH_3_C*H_2_*), 2.77/2.57 (s, 8 H; CH_2_C*H_2_*CN), 1.98 (s, 12 H; C*H_3_*CN), 1.45/1.21/0.94 ppm (d, 12 H; C*H_3_*CH_2_); ^13^C NMR (62.86 MHz, CDCl_3_, 25 °C, TMS): *δ*=146.0 (CH_3_*C*NCH_2_), 71.0/70.1 (CH_3_*C*H_2_), 30.7 (CN*C*H_2_CH_2_), 19.0/18.0 (*C*H_3_CH_2_), 16.7/13.1 ppm (*C*H_3_CN); ESI-MS: *m*/*z*: calcd: 583.2; found: 583.2 [Ti_2_**L^1^**_2_(OEt)_4_+Na]^+^.

[{Ti**L^2^**(OEt)_2_}_2_] (**3**): 1,5-Pentanedioxime (86 mg, 0.66 mmol) was dissolved in ethanol (3 mL), and Ti(OEt)_4_ (0.14 mL, 0.66 mmol) was added dropwise. The solution was stirred for 30 min at room temperature. Colorless crystals of **3** (112 mg, 61 %) were obtained after two weeks by slow evaporation of the solvent. ^1^H NMR (250 MHz, CDCl_3_, 25 °C, TMS): *δ*=4.34 (m, 4 H; CH_3_C*H_2_*), 3.64 (m, 4 H; CH_3_C*H_2_*), 2.50 (t, 8 H; CH_2_C*H_2_*CN), 1.84 (m, 4 H; CH_2_C*H*_2_CH_2_), 1.16/0.84 ppm (d, 12 H; C*H_3_*CH_2_); ^13^C NMR (62.86 MHz, CDCl_3_, 25 °C, TMS): *δ*=139.4 (*CH*NCH_2_), 71.7/68.2 (CH_3_*C*H_2_), 58.3 (CN*C*H_2_CH_2_), 27.9 (CH_2_*C*H_2_CH_2_), 22.2/18.0 ppm (*C*H_3_CH_2_); IR (ATR): $\tilde \nu $
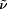
=2966 (C–H), 2919, 2859 (C–H), 1631 (C=N), 1437 (C–C), 1374 (C–O–Ti), 1114 (C–O), 1095, 1069, 1045 (C–O), 919, 887, 799 cm^−1^; ESI-MS: *m*/*z*: calcd: 555.1; found: 555.2 [Ti_2_**L^2^**_2_(OEt)_4_+Na]^+^.

[{Ti**L^3^**(OEt)_2_}_*x*_] (**4**): 1,3-Cyclohexanedioxime (94 mg, 0.66 mmol) was dissolved in ethanol (6 mL) and heated to reflux to dissolve the ligand, followed by dropwise addition of Ti(OEt)_4_ (0.14 mL, 0.66 mmol). The solution was stirred for 30 min at room temperature, while a red-brownish precipitate of **4** was formed, which was collected by filtration. ^13^C CP-MAS NMR: *δ*=143.0 (CH_2_*C*NCH_2_), 68.7 (O*C*H_2_CH_3_), 16.5 ppm (CH_2_*C*H_2_C, CH_2_*C*H_2_CH_2_, CN*C*H_2_CN, OCH_2_*C*H_3_); ^15^N CP-MAS NMR: *δ*=274.0 ppm.

[{Ti**L^4^**(OEt)_2_}_*x*_] (**5**): 1,4-Cyclohexanedioxime (94 mg, 0.66 mmol) was dissolved in ethanol (6 mL), heated to reflux to dissolve the ligand, followed by dropwise addition of Ti(OEt)_4_ (0.14 mL, 0.66 mmol). The solution was stirred for 30 min at room temperature, while a white precipitate of **5** was formed, which was collected by filtration. IR (ATR): $\tilde \nu $
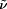
=2967 (C–H), 2926 (C–H), 1653 (C=N), 1437 (C–C), 1374 (C–O–Ti), 1359, 1327, 1122 (C–O), 996 (C–O), 943, 849, 824 cm^−1^.

[{Ti**L^5^**(O*i*Pr)_2_}_2_] (**6**): **L^5^**H_2_^11^ (214 mg, 0.66 mmol) was dissolved in 1,2-dichloroethane (3 mL), and Ti(O*i*Pr)_4_ (0.2 mL, 0.66 mmol) was added dropwise. The yellow solution was stirred for 20 min at room temperature. Removing the solvent under vacuum resulted in a solid residue. ^1^H NMR (250 MHz, CDCl_3_, 25 °C, TMS): *δ*=7.92 (s, 4 H; C*H*=N), 7.25 (d, 2 H; aryl–H), 7.14 (m, 2 H; aryl–H), 6.73 (m, 4 H; aryl–H), 4.63 (m, 2 H; (CH_3_)_2_C*H*), 4.47 (m, 2 H; (CH_3_)_2_C*H*), 3.18 (t, 8 H; CNC*H_2_*CH_2_), 1.23 (d, 24 H; (C*H_3_*)_2_CH), 1.03 ppm (m, 16 H; CH_2_C*H_2_*CH_2_); ^13^C NMR (62.86 MHz, CDCl_3_, 25 °C, TMS): *δ*=164.3 (aryl–*C*HN), 163.8 (aryl–CO), 134.2 (aryl–*C*H), 133.2 (aryl–*C*H), 122.2 (aryl–*C*–CHN), 118.8 (aryl–*C*H), 117.0 (aryl–*C*H), 77.9/76.0 ((CH_3_)_2_*C*H), 62.2 (CN*C*H_2_CH_2_), 31.3 (CH_2_*C*H_2_CH_2_), 26.9 (CH_2_*C*H_2_CH_2_), 26.5/25.3 ppm ((*C*H_3_)_2_*C*H); ESI-MS: *m*/*z*: calcd: 999.4; found: 999.5 [Ti_2_**L^5^**_2_(O*i*Pr)_4_+Na]^+^; calcd: 775.3; found: 775.4 [(Ti**L^5^**_2_)*i*PrOH+Na]^+^; calcd: 511.2; found: 511.2 [Ti**L^5^**(O*i*Pr)_2_+Na]^+^; calcd: 307.1; found: 307.1 [Ti(O*i*Pr)_4_+Na]^+^.

[{Zr**L^5^**(O*i*Pr)_2_}_2_] (**7**) was synthesized analogously by reacting Zr(O*i*Pr)_4_⋅*i*PrOH with **L^5^**H_2_ in toluene. ^1^H NMR (250 MHz, C_6_D_6_, 25 °C, TMS): *δ*=7.83 (s, 4 H; C*H*=N), 7.30 (d, 2 H; aryl–H), 7.19–7.05 (m, 4 H; aryl–H), 6.73 (m, 2 H; aryl–H), 4.63 (m, 4 H; (CH_3_)_2_C*H*), 3.42 (t, 8 H; CNC*H_2_*CH_2_), 1.86 (m, 8 H; CH_2_C*H_2_*CH_2_), 1.48 (d, 24 H; (C*H_3_*)_2_CH), 1.16 ppm (m, 8 H; CH_2_C*H_2_*CH_2_); ^13^C NMR (62.86 MHz, C_6_D_6_, 25 °C, TMS): *δ*=167.0 (aryl–*C*HN), 163.7 (aryl–CO), 134.6 (aryl–*C*H), 134.2 (aryl–*C*H), 122.4 (aryl–*C*–CHN), 120.3 (aryl–*C*H), 117.0 (aryl–*C*H), 71.4 ((CH_3_)_2_*C*H), 62.8 (CN*C*H_2_CH_2_), 31.3 (CH_2_*C*H_2_CH_2_), 26.7 ((CH_3_)_2_*C*H), 21.8 ppm (CH_2_*C*H_2_CH_2_); ESI-MS: *m*/*z*: calcd: 1095.3; found: 1095.3 [Zr_2_**L^5^**_2_(O*i*Pr)_4_+Cl]^−^; calcd: 1083.3; found: 1083.4 [Zr_2_**L^5^**_2_(O*i*Pr)_4_+Na]^+^.

[{Ti**L^6^**(O*i*Pr)_2_}_2_] (**8**): **L^6^**H_2_^11^ (228 mg, 0.66 mmol) was dissolved in 1,2-dichloroethane (3 mL) and Ti(O*i*Pr)_4_ (0.2 mL, 0.66 mmol) was added dropwise. The yellow solution was stirred for 20 min at room temperature. Removing the solvent under vacuum resulted in a solid residue. ^1^H NMR (250 MHz, CDCl_3_, 25 °C, TMS): *δ*=7.70 (s, 4 H; C*H*=N), 7.36 (d, 4 H; aryl–H), 7.00 (d, 4 H; aryl–H), 6.80–6.70 (m, 16 H; aryl–H), 4.51 (m, 4 H; (CH_3_)_2_C*H*), 4.00 (s, 8 H; CNC*H_2_*aryl), 1.26 ppm (d, 12 H; (C*H_3_*)_*2*_CH); ^13^C NMR (62.86 MHz, CDCl_3_, 25 °C, TMS): *δ*=165.8 (aryl–*C*HN), 164.0 (aryl–CO), 137.2 (aryl–C), 134.4 (aryl–*C*H), 133.7 (aryl–*C*H), 129.6 (aryl–*C*), 127.0 (aryl–*C*H), 122.2 (aryl–*C*–CHN), 118.9 (aryl–*C*H), 117.1 (aryl–*C*H), 78.0 ((CH_3_)_2_*C*H), 76.2 ((CH_3_)_2_*C*H), 63.6 (CN*C*H_2_CH_2_), 26.5 ((CH_3_)_2_*C*H), 25.1 ppm ((CH_3_)_2_*C*H); ESI-MS: *m*/*z*: calcd: 1039.4; found: 1039.3 [Ti_2_**L^6^**_2_(O*i*Pr)_4_+Na]^+^; calcd: 957.3; found: 957.3 [Ti_2_**L^6^**_2_(O*i*Pr)_3_]^+^.

[{Ti**L^6^**(OEt)_2_}_2_] (**9**) was synthesized analogously by treatment of **L^6^**H_2_ with Ti(OEt)_4_ in 1,2-dichloroethane. ^1^H NMR (250 MHz, CDCl_3_, 25 °C, TMS): *δ*=7.63 (s, 4 H; C*H*=N), 7.29 (d, 4 H; aryl–H), 7.00 (d, 4 H; aryl–H), 6.79–6.67 (m, 16 H; aryl–H), 4.38 (s, 8 H; CNC*H_2_*aryl), 4.14 (t, 8 H; CH_3_C*H_2_*), 1.21 (t, 6 H; C*H_3_*CH_2_), 0.99 ppm (t, 6 H; C*H_3_*CH_2_); ^13^C NMR (62.86 MHz, CDCl_3_, 25 °C, TMS): *δ*=166.1 (aryl–*C*HN), 163.4 (aryl–CO), 137.1 (aryl–C), 134.5 (aryl–*C*H), 133.5 (aryl–*C*H), 127.7 (aryl–*C*), 127.1 (aryl–*C*H), 122.2 (aryl–*C*–CHN), 119.0 (aryl–*C*H), 117.5 (aryl–*C*H), 71.8/70.5 (CH_3_*C*H_2_), 63.6 (CN*C*H_2_CH_2_), 18.9/18.5 ppm (*C*H_3_*C*H_2_); ESI-MS: *m*/*z*: calcd: 983.3; found: 983.4 [Ti_2_**L^6^**_2_(OEt)_4_+Na]^+^; calcd: 367.1; found: 367.2 [**L^6^**H_2_+Na]^+^.

[{Zr**L^6^**(O*i*Pr)_2_}_2_] (**10**) was synthesized analogously by reacting Zr(O*i*Pr)_4_⋅*i*PrOH with **L^6^**H_2_ in toluene. ^1^H NMR (250 MHz, CDCl_3_, 25 °C, TMS): *δ*=7.67 (s, 4 H; C*H*=N), 7.29 (d, 4 H; aryl–H), 6.95 (d, 4 H; aryl–H), 6.79–6.70 (m, 12 H; aryl–H), 6.59 (d, 4 H; aryl–H), 4.39 (m, 4 H; (CH_3_)_2_C*H*), 4.00 (s, 8 H; CNC*H_2_*aryl), 1.10 (d, 6 H; (C*H_3_*)_*2*_CH), 0.94 ppm (d, 6 H; (C*H_3_*)_*2*_CH); ^13^C NMR (62.86 MHz, CDCl_3_, 25 °C, TMS): *δ*=168.4 (aryl–*C*HN), 163.1 (aryl–CO), 137.1 (aryl–C), 134.6 (aryl–*C*H), 134.4 (aryl–*C*H), 129.6 (aryl–*C*), 127.3 (aryl–*C*H), 122.2 (aryl–*C*–CHN), 120.4 (aryl–*C*H), 117.0 (aryl–*C*H), 71.3 ((CH_3_)_2_*C*H), 63.0 (CN*C*H_2_CH_2_), 26.6 ppm ((*C*H_3_)_2_*C*H); ESI-MS: *m*/*z*: calcd: 1135.3; found: 1135.2 [Zr_2_**L^6^**_2_(O*i*Pr)_4_+Cl]^−^.
